# An old weapon with a new function: PIWI-interacting RNAs in neurodegenerative diseases

**DOI:** 10.1186/s40035-021-00233-6

**Published:** 2021-03-08

**Authors:** Xiaobing Huang, Garry Wong

**Affiliations:** Centre of Reproduction, Development and Aging, Faculty of Health Sciences, University of Macau, Macau, 999078 S.A.R. China

**Keywords:** piRNA, piRNA biogenesis, Transposable element, Neurodegenerative disease, H3K9me3

## Abstract

PIWI-interacting RNAs (piRNAs) are small non-coding transcripts that are highly conserved across species and regulate gene expression through pre- and post-transcriptional processes. piRNAs were originally discovered in germline cells and protect against transposable element expression to promote and maintain genome stability. In the recent decade, emerging roles of piRNAs have been revealed, including the roles in sterility, tumorigenesis, metabolic homeostasis, neurodevelopment, and neurodegenerative diseases. In this review, we summarize piRNA biogenesis in *C. elegans*, *Drosophila*, and mice, and further elaborate upon how piRNAs mitigate the harmful effects of transposons. Lastly, the most recent findings on piRNA participation in neurological diseases are highlighted. We speculate on the mechanisms of piRNA action in the development and progression of neurodegenerative diseases. Understanding the roles of piRNAs in neurological diseases may facilitate their applications in diagnostic and therapeutic practice.

## Background

PIWI-interacting RNAs (piRNAs) are a class of small non-coding RNAs widely existing in nematodes, insects, zebrafish, *Drosophila*, *Aplysia* and mammals that regulate transposon and gene expression [1]. There are several differences between microRNA (miRNA) and piRNA. First, piRNA is processed from a single-stranded precursor transcribed from a piRNA cluster and the biogenesis does not require Drosha or Dicer proteins. In contrast, miRNA originates from a double-stranded precursor and the primary miRNA transcript is processed by Drosha to generate a pre-miRNA, which is then further modified by Dicer to become a mature miRNA [2]. Second, miRNA and piRNA differ in size. miRNA is 21–25-nucleotide (nt) long, while the piRNA is 24–32 nt in most species with exception in *C. elegans* (21 nt) [3]. Third, miRNA and piRNA have different chemical structures. miRNA is a small duplex RNA with a 5′ monophosphate and a 2′,3′ hydroxyl, 2-nt overhanging 3′ end, while piRNAs typically have a uridine at the 5' end and 2′-*O*-methylation at the 3′ end [2]. In addition, piRNAs use the ping-pong cycle to amplify the piRNA signal in *Drosophila* and mice, or recruit an RNA-dependent RNA polymerase to synthesize massive secondary small interfering RNAs (22G-RNAs) during the biogenesis process, whereas miRNAs are transcribed from the genome without amplification. Finally, most miRNAs degrade mRNA directly, while piRNAs can cleave mRNA directly or induce transcriptional silencing by recruiting histone 3 lysine 9 tri-methylation (H3K9me3) and histone methyltransferase to induce histone modification or DNA methylation [4].

The role of piRNAs in silencing unwanted germline transcripts and maintaining genome integrity has been studied in detail, but their role in somatic cells is less clear. In recent years, piRNAs have been shown not only to protect germline genome against transposon mobilization to maintain the genome stability, but also to participate in neuronal growth and development [5], metabolic homeostasis [6], self-renewal of stem cells [7, 8], cancer [9, 10], and processes of neurodegenerative diseases [11–15]. Moreover, the number of piRNAs discovered is increasing rapidly. Until 2018, there has been discovery of 8,438,265 piRNAs in humans, 68,054,594 in mice, 41,950,613 in *Drosophila* and 28,219 in *C. elegans* in the piRBase [16].

In this review, we follow the life of piRNAs from their biogenesis to their canonical role in silencing transposons and to their latest roles in neurological function and dysfunction. We further present current knowledge on piRNA function in different animal model systems and recent work demonstrating the presence and putative functions of piRNAs in neuronal tissues. Finally, we provide perspectives on the roles of piRNAs in neurodegenerative diseases based on the current studies. While the translation of piRNA research remains ongoing, this review would provide an organized and informative foundation for current and future efforts.

## Main text

### piRNA biogenesis in *C. elegans*, *Drosophila* and mice

piRNAs originate from either piRNA clusters that are located in the genome, or from RNA transcripts of active transposable elements. The biogenesis of piRNA has been well studied in *C. elegans* and *Drosophila* due to their simplified genetic manipulation. After being transcribed from a piRNA cluster, piRNAs are loaded into the PIWI Argonaute proteins to form an RNA-induced silencing complex (RISC). This complex recognizes its targets through base pairing, which does not require perfect matching for its target transcript [17]. Structurally, the PIWI protein includes three domains: the N-terminal PAZ domain, which binds to the 3' end of the guide RNA; the middle MID domain, which binds to the 5' end of the RNA; and the C-terminal domain that contains an endonuclease which cleaves the transcript [18]. The PIWI Argonaute proteins are highly conserved from *C. elegans*, *Drosophila*, mice, to humans. In *C. elegans*, there is only one PIWI protein, PRG-1, which combines with piRNA to recognize non-self-transcripts [19, 20]. In *Drosophila*, there are 3 PIWI proteins, Piwi, Aubergine (Aub) and Argonaute 3 (Ago3) [21]. In mice, there are also 3 PIWI homologs, PIWIL1 (MIWI), PIWIL2 (MILI), and PIWIL4 (MIWI2) [22]. In humans, there are 4 PIWI homologs, PIWIL1 (HIWI1), PIWIL2 (HILI), PIWIL3 (HIWI3), and PIWIL4 (HIWI2) (Table 1) [38]. PIWI proteins play a crucial role in maintaining the activity of piRNAs. Mutations of the PIWI gene can cause sterility not only in *C. elegans*, *Drosophila*, and mice, but also in humans [28, 39–41]. Here, we discuss the piRNA biogenesis in *C. elegans*, *Drosophila*, and mice, with a particular emphasize on *C. elegans*, as detailed reviews on piRNA biogenesis in *Drosophila* and mice have been well summarized elsewhere [2, 4, 23, 37, 42]. The comparsion of piRNA biogenesis among *C. elegans*, *Drosophila*, mice and humans is shown in Table 1.

**Table 1:** A comparison of piRNA biogenesis among *C. elegans*, *Drosophila*, mice and humans

	***C. elegans***	***Drosophila***	Mouse	Human	Reference
Size	21 nt	23-29 nt	24-30 nt	26-32 nt	[23–26]
Structure	5′ Uridine 3′ 2′-O-methylation	5′ Uridine 3′ 2′-O-methylation	5′ Uridine 3′ 2′-O-methylation	5′ Uridine	[4, 25–27]
PIWI proteins	PRG-1	Piwi Aub Ago3	MIWI (PIWIL1) MILI (PIWIL2) MIWI2 (PIWIL4)	HIWI (PIWIL1) HILI (PIWIL2) HIWI3 (PIWIL3) HIWI2 (PIWIL4)	[19–22, 28]
Transcription loci	Type I loci, Type II loci, mainly from chromosome IV	*flamenco* loci, *42AB* loci, distributed across all chromosomes	Distinct intergenic loci, distributed across all chromosomes	Distributed across 22 autosomal chromosomes and X chromosome	[24, 26, 29–32]
Amplification	RdRp	Ping-pong cycle	Ping-pong cycle	Ping-pong cycle	[33–36]
Biogenesis factor	FKH transcription factors, TOFU genes, PRG-1, EGO-1	RDC complex, MitoPLD/Zuc, TDRD proteins, PIWI proteins	A-MYB transcription factor, MitoPLD/Zuc, TDRD proteins, PIWI proteins	PIWI proteins	[23, 33, 37]
Downstream effects	RNA degradation, histone modification, 22G-RNA biogenesis	RNA degradation, histone modification	RNA degradation, histone modification, DNA methylation	RNA degradation, histone modification, DNA methylation	[23, 33, 37]

Abbreviations: *RdRp* RNA-dependent RNA polymerases, *RDC complex* Rhino-Deadlock-Cutoff complex, *FKH* Fork head

### piRNA biogenesis in *C. elegans*

**Fig. 1:**
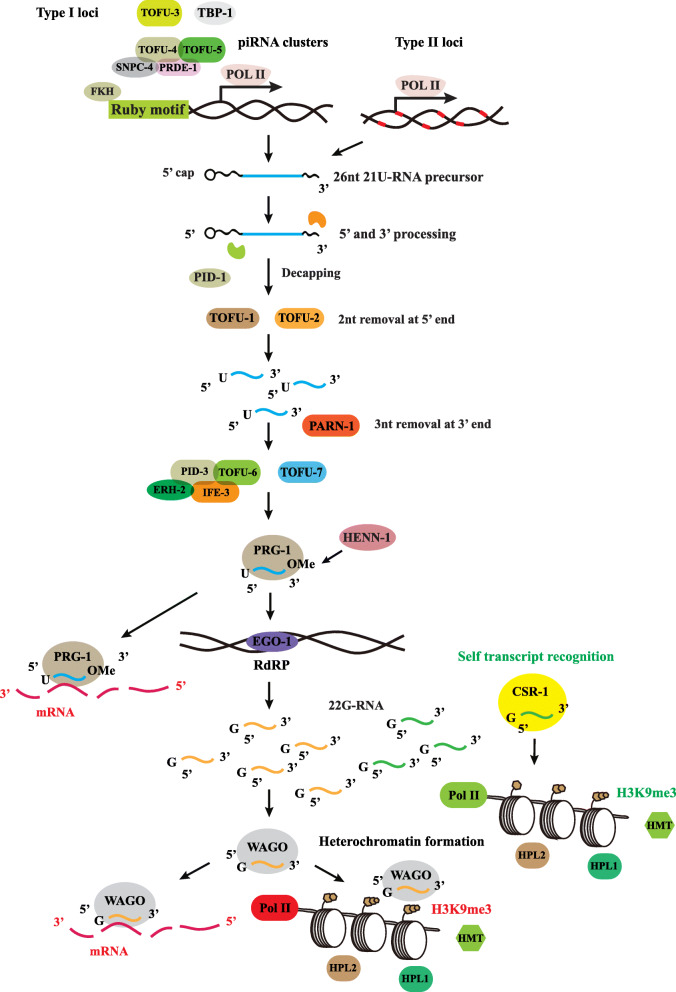
piRNA biogenesis in *C. elegans*. The fork head (FKH) family transcription factors recognize the 8-nt Ruby motif, then the upstream sequence transcription complex (containing PRDE-1, TOFU-4, TOFU-5, and SNPC-4) binds to piRNA gene promoter and recruits TOFU-3 and TBP-1 to transcribe the 21 U-RNA precursors (26-nt single-stranded RNA transcripts). The 21 U-RNA precursors are exported to cytoplasm, 2 nt is removed by TOFU-1 and TOFU-2 at the 5′ end, and the PETISCO complex (containing PID-3, ERH-2, TOFU-6, and IFE-3) and TOFU-7 enhance maturation. The PICS complex refers to TOFU-6, PICS-1, ERH-2, PID-1 and TOST-1. PARN-1 removes 3 nt in the 3′ end and 2′-O-methyl is methylated by HENN-1 to generate the mature 21 U-RNAs (21-nt single-stranded RNA transcripts). The 21 U-RNAs combine with PRG-1 to form RISC to target and degrade transposons or mRNA directly. Additionally, the piRNAs/PRG-1 complex recruits RdRP to produce massive secondary siRNAs (22G-RNAs) by EGO-1. These 22G-RNAs are loaded into the Argonaute protein and cause post-transcriptional silencing by cleaving transposon or mRNA directly or recruiting H3K9me3, HPL-1 and HPL-2 to induce transcriptional silencing. The piRNA/PRG-1 complex can recognize and differentiate self-transcripts from the non-self-component. The 22G-RNAs load into the CSR-1 Argonaute protein, and once 22G-RNAs/CSR-1 recognize the self-transcript, it will protect mRNA from silencing. The balance between self-recognition and non-self-distinguishing determines the outcome of gene expression in worms

In *C. elegans*, piRNA is referred to as 21 U-RNA due to the precise 21-nt length and 5′-end uridine [29]. piRNA is transcribed from two types of loci. The type I loci have a conserved 8-nt Ruby motif (CTGTTTCA) or a YRNT motif. These motifs are located in chromosome 4, mainly reside in introns and intergenic regions and are transcribed by RNA polymerase II [24, 43]. The type II loci are distributed throughout the genome and have no apparent upstream motif. The type II 21 U-RNA can be transcribed by RNA polymerase II bidirectionally from its transcription start-site. They are localized throughout the genome and are decentralized [24, 29]. The transcription of type I piRNAs has been well-studied and constitutes ~ 95% of the mature 21 U-RNA population, while the type II 21 U-RNAs only have a minimal contribution [44]. In the following, we will mainly focus on the type I piRNA transcription (Fig. 1).

When piRNAs are transcribed, the Fork head family transcription factors recognize the 8-nt Ruby motif [45]. The upstream sequence transcription complex (USTC), which contains PRDE-1, TOFU-4, TOFU-5 and SNPC-4, binds to the piRNA gene promoter to transcribe the 21 U-RNA precursor [46]. USTC also recruits other factors such as TOFU-3 and TBP-1 as additional cofactors for the piRNA transcription [46]. Each locus can produce an independent 26-nt single-stranded RNA transcript.

The 26-nt 21 U-RNA precursor is then exported to the cytoplasm, where it is further decapped by TOFU-1 and TOFU-2 to remove 2 nt at the 5′ end [29, 47]. TOFU-6 and TOFU-7 facilitate the maturation of 21 U-RNA [29, 47]. PARN-1, a conserved 3′-to-5′ exo-RNase, trims the 3′ end and removes 3 nt at the 3′ end, resulting in a 21-nt transcript [48]. Then the 21 U-RNA is loaded into PRG-1 and methylated by HENN-1 to carry a 2′-O-methyl modification, generating mature 21 U-RNA [49]. The mature 21 U-RNA combines with PRG-1 to form an RISC to target and degrade a transposon or mRNA through sequence complementarity [29, 33]. PRG-1 is the unique PIWI protein in *C. elegans* and mutations of *prg-1* can lead to a reduction of brood size and a temperature-sensitive sterile phenotype [50], suggesting that the piRNAs mainly function in the germline to maintain genome stability.

The piRNAs/PRG-1 complex recruits the RNA-dependent RNA polymerases to produce massive secondary siRNAs (22G-RNAs) by EGO-1 [34]. These 22G-RNAs are loaded into the Argonaute protein and cause post-transcriptional gene silencing by directly cleaving transposons or mRNA to induce transcriptional gene silencing [50, 51]. Once the 22G-RNAs/WAGO complex enters the nucleus, it can recruit histone methyltransferase like HPL-1 or HPL-2 to add the repressive chromatin marks histone 3 lysine 9 di-methylation (H3K9me2)/ H3K9me3 at the genomic site, after which the heterochromatin formation can silence the Pol II activity [50, 52].

The piRNA/PRG-1 complex does not require a perfect match for its target transcript except for positions 2–8 of the 21 U-RNAs [17]. Its potential target range is broad; therefore, piRNAs require an additional mechanism to recognize the self-transcript from the non-self-component. Furthermore, the 22G-RNAs can load into the CSR-1 Argonaute protein. Once the 22G-RNAs/CSR-1 complex recognizes the self-transcript, it will protect mRNA from being silenced [53]; hence, the 22G-RNAs/CSR-1 acts as a negative form against the activity of 22G-RNAs/WAGO [53]. How piRNA is able to distinguish between “self” and “non-self” components remains a mystery. Research has shown that PRG-1 scans for foreign sequences and the Argonaute proteins WAGO-1 and CSR-1 serve as an epigenetic memory of “self” and “non-self” transcripts to recognize mRNA. The balance between self- and non-self-recognition determines the outcome of gene expression in worms [54, 55].

Additional factors for piRNA biogenesis have been discovered in *C. elegans*, including PID-1, which might be a potential nuclear import and export factor that guides the 21 U-RNA precursors to the site where they will be further modified to become mature 21 U-RNA [56]. Recently, a new complex PETISCO (PID-3, ERH-2, TOFU-6, and IFE-3 small RNA complex) has been identified to have both potential 5′-cap and 5′-phosphate RNA-binding domains, suggesting that this complex can interact with capped 21 U precursor RNAs to enhance the maturation of 21 U-RNAs [57]. Furthermore, Zeng et al. using functional proteomics identified a piRNA biogenesis and chromosome segregation complex, PICS (containing TOFU-6, PICS-1, ERH-2, PID-1 and TOST-1), which is required for 21 U-RNA maturation and exhibits dynamic localization during chromosome segregation [58]. The factors involved in piRNA biogenesis in *C. elegans* and their functions are summarized in Table 2.

**Table 2:** Factors involved in piRNA biogenesis in *C. elegans*

Protein symbol	Type	Function	Phenotype of mutants	Human orthologue	Reference
FKH-3	ForKHead transcription factor	Recognizing the Ruby motif; involved in type I 21 U-RNA biogenesis	N/A	N/A	[24, 45]
FKH-4	ForKHead transcription factor	Recognizing the Ruby motif; involved in type I 21 U-RNA biogenesis	N/A	N/A	[24, 45]
FKH-5	ForKHead transcription factor	Recognizing the Ruby motif; involved in type I 21 U-RNA biogenesis	N/A	N/A	[24, 45]
UNC-130	UNCoordinated 130	Binding to 21 U-RNA upstream regulatory sequence; required for piRNA transcription	N/A	FOXD4	[59]
SNPC-4	Small nuclear RNA activating protein complex	Binding to piRNA clusters; required for global piRNA expression	Lethal or sterile	SNAPC4	[59]
PRDE-1	Nuclear factor	Required for production of piRNA precursors from the Ruby motif; recruiting RNA polymerase II to the FKH motif	Mrt	N/A	[29, 60]
TOFU-3	Twenty-One-u Fouled Ups	Required for the production or stability of 21 U-RNA precursors; a SUMO-related protease; interacting with HP1a and regulating its localization to pericentric heterochromatin	Mrt	N/A	[29, 47]
TOFU-4	Twenty-One-u Fouled Ups	Required for the production or stability of 21 U-RNA precursors	Mrt	N/A	[29, 47]
TOFU-5	Twenty-One-u Fouled Ups	Required for the production or stability of 21 U-RNA precursors; having a DNA-binding domain structurally related to that of Myb and found in various transcription regulatory factors	Lethal or sterile	N/A	[29, 47]
TBP-1	TATA-box-binding protein	An additional factor required for TOFU-5 binding to piRNA clusters	N/A	TBP TBPL2	[46]
PID-1	piRNA-induced silencing-defective 1	A potential nuclear import and export factor	Lethal	N/A	[56]
TOFU-1	Twenty-One-u Fouled Ups	Required for precursor RNA processing after its production	Mrt	N/A	[29, 47]
TOFU-2	Twenty-One-u Fouled Ups	Required for precursor RNA processing after its production	Mrt	N/A	[29, 47]
PARN-1	RNase	Required for piRNA 3′ end trimming and having a 3′-to-5′ exo-RNase activity	Mrt	PARN PNLDC1	[48]
TOFU-6	Twenty-One-u Fouled Ups	A component of PETISCO; required for 21 U biogenesis	N/A	N/A	[29, 47]
TOFU-7	Twenty-One-u Fouled Ups	Required for precursor RNA processing after its production	N/A	N/A	[29, 47]
HENN-1	HEN methyltransferase 1	Required for precursor RNA processing after its production	Sterile	HENMT1	[61, 62]
PID-3 (PICS-1)	piRNA induced silencing defective	A component of PETISCO; required for 21 U biogenesis; interacting with 21 U-RNA precursor	Mes	N/A	[57]
ERH-2	Enhancer of rudimentary homolog	A component of PETISCO; required for 21 U biogenesis; interacting with 21 U-RNA precursor	Mes	ERH	[57]
IFE-3	Homologs of eIF4E	A component of PETISCO; required for 21 U biogenesis; binding to the 5′ cap of the 21 U precursor transcripts	Mes	EIF4E EIF4E1B	[57]
EGO-1	Argonaute	RNA-dependent RNA polymerase	Sterile	N/A	[34, 63]
PRG-1	PIWI protein	21 U-RNA binding activity; combining with piRNA to form RISC	Mrt	PIWIL1 PIWIL2 PIWIL3 PIWIL4	[19, 20]
CSR-1	Argonaute	Self- and non-self-transcript discrimination	Mrt	N/A	[54, 55]
TOST-1	Twenty-one U pathway antagonist	Inhibiting 21 U-driven silencing	Mes	N/A	[57]
EKL-1	Enhancer of *ksr-1* Lethality	Encoding a Tudor-domain protein required for RNAi; transgene silencing; transgene-mediated cosuppression in the germline	N/A	N/A	[47, 64]
DRH-3	Dicer related helicase	A core component of the RdRp complex and essential for 22G-siRNA biogenesis	N/A	DHX58	[65]
RRF-1	Argonaute	RNA-dependent RNA polymerase	N/A	N/A	[66]
RDE-2	RNAi defective	Interacting with MUT-7; required for efficient RNAi	N/A	N/A	[67]
MUT-7	Mutator	Encoding a putative exoribonuclease; required for efficient RNAi	N/A	EXD3	[67]
RDE-8	RNAi defective	Cleaving target mRNAs to mediate silencing; generating mRNA fragments to serve as template for RdRp	N/A	N/A	[68]
MUT-16	Mutator	Required for the accumulation of 22G-siRNA	N/A	N/A	[69]
SIMR-1	Tudor domain protein	Mediating the production of secondary siRNAs by the mutator complex	Mrt	N/A	[70]
HRDE-1	Nuclear Argonaute	Germline nuclear RNAi	Mrt	N/A	[71]
NRDE-3	Nuclear Argonaute	Somatic nuclear RNAi	Mrt	N/A	[71]
MET-2	H3K9me1/H3K9me2 methyltransferases	Required for 22G-siRNA formation	Mrt	SETDB1 SETDB2	[72]
SET-25	H3K9me3 methyltransferase	Heterochromatin maintenance, required for maintenance of piRNA-induced RNAi	N/A	N/A	[72]
SET-32	H3K9me3 methyltransferase	Silencing establishment; required for multigenerational inheritance of environmental RNAi and piRNA silencing	Mrt	N/A	[73]
HPL-1	Heterochromatin protein 1	Binding with H3K9me2/H3K9me3; required for piRNA inducetion of heritable small RNAs	Sterile	CBX1 CBX3 CBX5	[74]
HPL-2	Heterochromatin protein 2	Binding with H3K9me2/H3K9me3; required for piRNA inducetion of heritable small RNAs	Sterile	CBX3	[75]
WAGO	Argonaute	Argonaute protein in *C. elegans* required for 22G-siRNA after RNA-dependent RNA polymerase activity	N/A	AGO1 AGO2 AGO3 AGO4	[76]

Abbreviations: *Mrt* mortal germline, *Mes* maternal-effect sterility, *N/A* not available

### piRNA biogenesis in *Drosophila*

**Fig. 2:**
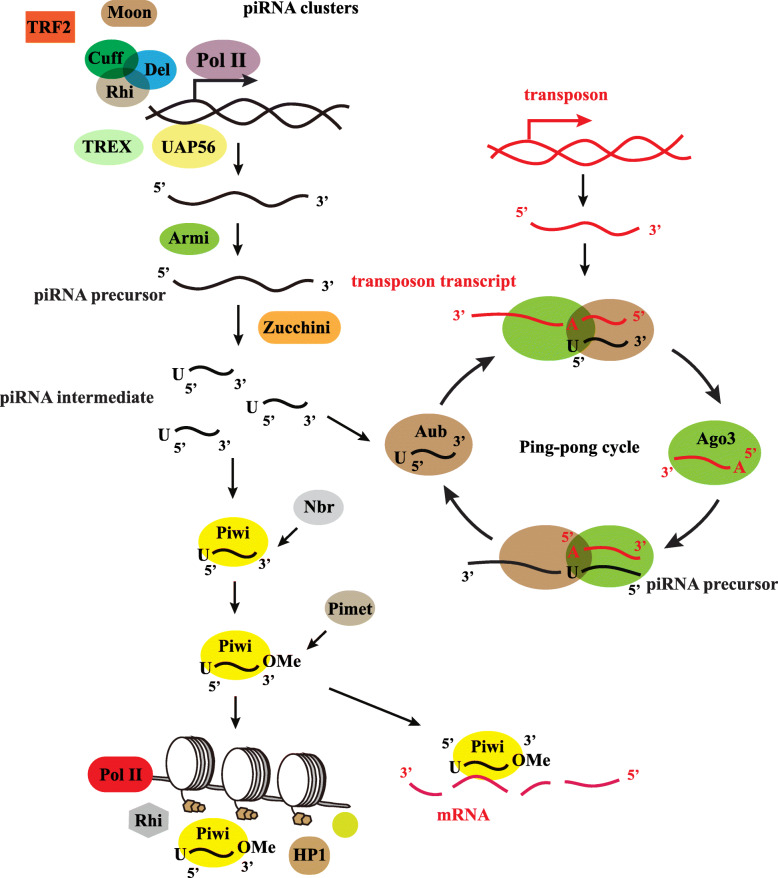
piRNA biogenesis in *Drosophila*. piRNAs are transcribed from piRNA clusters as a single-stranded transcript. RNA Pol II and the Rhino-Cutoff-Deadlock (RDC) complex are recruited to the piRNA cluster, and Moon facilitates the initiation of transcription and inhibits the H3K9me3 mark by interacting with the RDC complex and TRF2. TREX prevents the nascent RNA and DNA from forming the R-loop with UAP56 to suppress the splicing of piRNA precursors. After piRNA transcription, the single-stranded transcript is exported to the cytoplasm and the secondary structure is resolved by the RNA helicase Armitage. Then MitoPLD/Zucchini cleaves the piRNA precursor into piRNA intermediates. The pre-mature piRNAs are loaded into the Piwi protein and trimmed by Nibbler at the 3′ end. The newly formed 3′ terminal end is methylated by DmHen1/Pimet methyltransferase and the 2′-O-methylate is added to the 3′ end of piRNAs to produce the mature Piwi-piRNA complexes. The Piwi-piRNA complexes can degrade mRNA directly or recruit HP1a, Su (var)3–9 and H3K9me2/H3K9me3 to the genomic site to silence the activity of RNA polymerase II. The Aub-piRNA complex can trigger the ping-pong cycle in cytoplasm together with Ago3 to produce secondary piRNAs. In the ping-pong cycle, Ago3 combines with sense transposon transcripts and acts *via* sequence complementarity to match with antisense transposon transcripts. After several cycles, the Aub-piRNA complex consumes the sense transposon transcripts to silence transposons in germline cells

In *Drosophila,* the piRNA biogenesis occurs through a primary piRNA pathway and a secondary ping-pong cycle for amplification [4]. In somatic cells, piRNAs only go through a primary processing pathway, because the ping-pong cycle occurs exclusively in germline cells [4]. piRNAs are transcribed from piRNA clusters such as the *flamenco* (*flam*) locus or the *42AB* locus as a single-stranded transcript [30, 31]. This transcription requires several proteins. First, RNA Pol II is recruited to the piRNA cluster. Next, the transcription is assisted by a Rhino-Cutoff-Deadlock (RDC) complex. Within this complex, Rhino prevents inaccurate splicing and represses the use of canonical cleavage and polyadenylation sequence motifs [77]. The Cutoff protein inhibits premature termination and prevents the degradation of the precursor [78]. The Deadlock protein is a linker that recruits Cutoff and links it to Rhino to form the RDC complex [79]. Then, Moonshiner, a transcription factor IIA subunit, facilitates the initiation of transcription and inhibits the H3K9me3 mark by interacting with the RDC complex and TRF2 [80]. TREX prevents the nascent RNA and DNA to form the R-loop [81]. UAP56 suppresses the splicing of piRNA precursors (Fig. 2) [82].

After piRNA transcription, the single-stranded transcript is exported to the cytoplasm and the secondary structure is resolved by the RNA helicase Armitage. After despiralization, the piRNA precursor is processed by MitoPLD/Zucchini, an endonuclease located on the surface of mitochondria. MitoPLD cleaves the piRNA precursor into piRNA intermediates [83]. The pre-mature piRNAs are loaded into the Piwi protein and trimmed by a 3′-to-5′ exonuclease of Nibbler at the 3′ end. Meanwhile, the newly formed 3′-terminal end is methylated by the DmHen1/Pimet methyltransferase and the 2′-O-methylate is added to the 3′ end of piRNAs to produce the mature Piwi-piRNA complex [84–86]. The Piwi-piRNA complex can degrade mRNA directly. Once these complexes are exported to the nucleus, they can recruit histone methyltransferases HP1a, Su (var)3–9 and H3K9me2/H3K9me3 to the genomic site to silence the activity of RNA polymerase II (Fig. 2) [87].

The Aub-piRNA complex, together with Ago3, can trigger the ping-pong cycle in the cytoplasm to produce secondary piRNAs. In the ping-pong cycle, Ago3 combines with sense transposon transcripts (derived from the transposon) and acts *via* sequence complementarity to match with the antisense transposon transcripts (derived from piRNA clusters) [35]. The Aub-piRNA complex cleaves sense transposon transcripts through its slicer activity at positions 10 nt and 11 nt from the 5′ ends of their target RNAs [35]. After several cycles, the Aub-piRNA complex consumes most of the sense transposon transcripts to silence transposons in germline cells. The most important features of the ping-pong cycle are that the Aub-piRNAs show an antisense and 1 U bias, while the secondary piRNAs loaded onto Ago3 show 10 nt complementarity at their 5′ ends and possess a 10A bias [30, 88]. Other factors like the Tudor domain-containing (TDRD) proteins and Tudor superfamily members are involved in piRNA biogenesis. There are 11 TDRD proteins in *Drosophila*, including Tudor, Partner of piwi, Spindle-E, Qin/Kumo, Tejas, Yb, Brother of Yb, Sister of Yb, dSETDB1, Krimper, and Vreteno [3]. These proteins are mainly involved in splicing, loading, and processing of piRNA. Details of most of these proteins and their functions can be found elsewhere [3, 4].

### piRNA biogenesis in mice

**Fig. 3:**
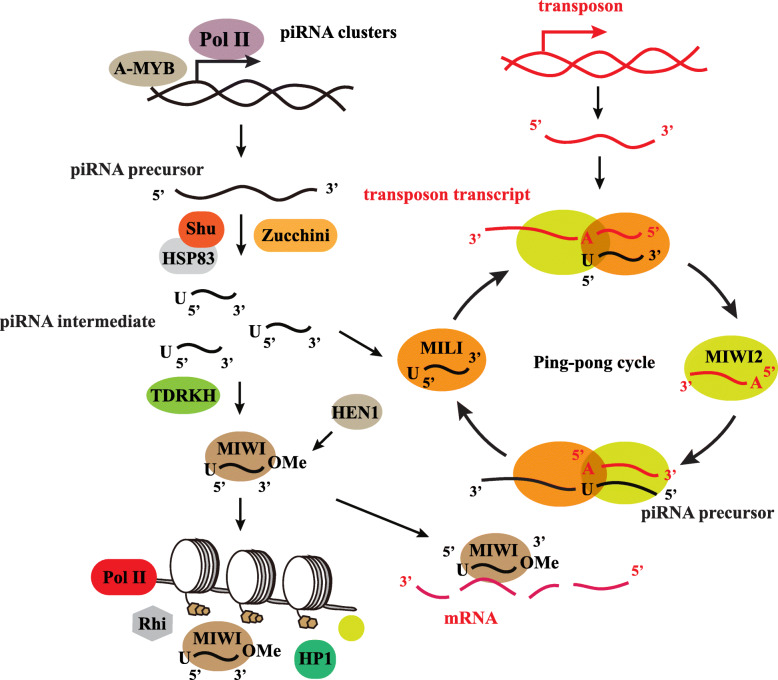
piRNA biogenesis in mice. The A-MYB transcription factor initiates the transcription of precursor piRNAs from piRNA clusters. The long single-stranded transcript is cut by MitoPLD/Zucchini into piRNA intermediates, with the help of Shutdown and Hsp83 co-chaperones. The piRNA intermediates are incorporated into MIWI protein. The 3′ end is trimmed by exonuclease TDRKH and the 2′-O-methylate is added by methyltransferase Hen1. The ping-pong cycle then generates secondary piRNAs and amplifies both primary and secondary piRNAs. MIWI2 is localized to the nucleus upon piRNA loading. MILI combines with piRNA to trigger secondary piRNA biogenesis. MILI cuts the complimentary mRNA, which then combines with MIWI2. MIWI2 undergoes a similar process that targets the piRNA precursor and cleaves piRNA. The MIWI2-piRNA complex can translocate into the nucleus to recruit modification enzymes and causes DNA methylation or histone modification to silence gene expression

Similar piRNA biogenesis processes have been observed in *Drosophila* and mice. In mice, the A-MYB transcription factor initiates the transcription of precursor piRNAs from piRNA clusters. The long single-stranded transcript is cut by MitoPLD/Zucchini into piRNA intermediates with the help of Shutdown and heat shock protein 83 co-chaperones [89]. The piRNA intermediates are incorporated into the PAZ domain of the MIWI protein [90]. After loading into the MIWI protein, a uridine residue is added to the 5′ end, the 3′ end is trimmed by the exonuclease TDRKH, and 2′-O-methylation is added by Hen1 methyltransferase (Fig. 3) [4].

The primary biogenesis process produces the initial piRNAs. The ping-pong cycle then generates secondary piRNAs and amplifies both primary and secondary piRNAs. MIWI2 associated with secondary piRNAs is localized to the nucleus upon piRNA loading [33]. MILI combines with piRNA to trigger secondary piRNA biogenesis. MILI cuts the complimentary mRNA between 10 nt and 11 nt from the 5′ end of the piRNA, which leads to the RNA product containing an adenine residue at the 10th position, then the piRNA binds with MIWI2 [33]. MIWI2 undergoes a similar process that targets the piRNA precursor and cleaves piRNA that contains a uridine residue at the 5′ end and is trimmed at the 3′ end. The MIWI2-piRNA complex can translocate into the nucleus to recruit modification enzymes and cause DNA methylation or histone modification [91]. Many additional proteins are involved in the piRNA biogenesis pathway, including the TDRD proteins (TDRD1, TDRD2, TDRD4, TDRD5, TDRD6, TDRD7, and TDRD9), GASZ, GPAT2, and MOV10L1. Fu et al. have summarized most of these proteins and their functions elsewhere [92].

### piRNAs silence transposable elements

The maintenance of genomic integrity over generations is a major selection force in animals. Transposable elements are mobile elements and have propensity to cause ectopic recombination, therefore being the risk factor against genomic integrity [93]. Transposons can cause insertional mutagenesis and/or turn on or off nearby genes [94]. Some of the transposon insertion changes can affect the splicing pattern while others may cause gene duplication, deletion, translocation or inversion [95], which are detrimental for the organism. There are two types of transposon. The class I transposon contains retrotransposons and the class II contains DNA transposon elements [96]. The difference between these two transposons is that the class I transposons can mobilize *via* a “copy and paste” mechanism, through which an RNA intermediate is reverse transcribed into cDNA and the cDNA subsequently integrates into the genome, while the class II transposons mobilize through a “cut and paste” mechanism, through which a DNA intermediate is cut from its anterior location and integrates into a new position in the genome [97]. The DNA transposon is not active due to the cumulative mutations and truncations in the genome. The common DNA transposons include *hAT*, *MuDR*, *piggyBac*, and *Tc1/mariner* in the human genome [98].

Retrotransposons form the majority of transposable elements in the genome [99], and can be further classified into long-terminal repeats (LTRs) and non-long-terminal repeats (non-LTRs). The LTR elements, also called endogenous retroviruses, constitute almost 8% of the human genome [100]. The most common type of LTR includes the family of human endogenous retroviruses (HERV) such as HERV-E, HERV-W, and HERV-FRD. The most active type of transposon is the non-LTR that includes the long-interspersed elements (LINEs) (constituting 20% of human genome sequence) and short-interspersed element (constituting 10% of human genome sequence) [93]. Other types of non-LTR include *Alu*, *MIR*, and *SVA* that are also highly expressed in human cells.

Transposons are active in the genome and might cause mutations in cells. At least 124 diseases are known to result from transposable element insertions [101], and 118 human disease mutations are due to the retrotransposons [94]. The piRNA pathway serves as the major protector against the transposon, while a mutation in the PIWI gene could lead to transposable element derepression, genomic instability, and infertility [102]. Here, we describe several mechanisms through which piRNAs act to block retrotransposon activity.

### RNA degradation

piRNAs bind to PIWI protein and form a complex, where the PIWI C-terminal domain has endonuclease activity that can cleave transcripts directly. In mice and *Drosophila*, the ping-pong cycle degrades most of the transcripts of the transposon. In mice, the initiation of secondary piRNA biogenesis is guided by the endonucleases of the PIWI protein [88]. piRNAs can combine with MILI to form a complex that cuts the complimentary mRNA by endonucleases. New transcripts can combine with MIWI2 to form the new piRNA-MIWI2 complex and the ping-pong cycle continues (Fig. 3). To explore the role of PIWI-catalyzed endonucleolytic activity, De Fazio et al. designed point mutations in mice, which resulted in substitution of the second aspartic acid to alanine in the DDH catalytic triad of MILI and MIWI2 [103]. The results showed that the transposon piRNA amplification was eliminated in the mice, resulting in reduced piRNA bound with MIWI2 ribonuclear particles, suggesting that the PIWI complex cleavage of the transposon is necessary for the secondary piRNA biogenesis [103].

The same process of transposable element degradation also occurs in *Drosophila*. In the piRNA biogenesis process in *Drosophila*, Aub and Ago3 act as partners to defend against transposons. During the ping-pong cycle to amplify piRNA, Aub is loaded with primary piRNAs that are derived from piRNA clusters and are antisense to transposon transcripts [104]. Once the Aub-piRNA complex recognizes the target transcripts through sequence complementarity, Aub cleaves the target transcript and this not only destroys the transposon, but also generates a new piRNA precursor, which can be incorporated into Ago3 [104]. The Ago3-bound piRNAs are sense to the transposable element, so these piRNAs can promote the production of a new round of piRNAs that are derived from the piRNA cluster, by recognizing complementary cluster transcripts. The piRNA-complementary transcript complex is then cleaved and the new piRNAs are incorporated into Aub (Fig. 2) [104]. The transposons are degraded in this ping-pong cycle.

### DNA methylation

DNA methylation is the main mechanism to silence transposons in mammals. In mice, it is hypothesized that MIWI2 recruits a DNA methylation apparatus to silence the active transposons. The loss of MIWI2 causes similar phenotypes as that by *Dnmt3L* (DNA methyltransferase 3-Like) knockout in mice. MIWI2 uses antisense piRNAs as a guide to detect the initial transposable element transcripts and induces de novo DNA methylation activity or recruits H3K9me3 to establish the repressive chromatin landscape to silence transposons, particularly targeting the active long interspersed nuclear element1 (LINE1) elements [105]. MILI and MIWI2 have different functions in transposable element repression. The knockout of MIWI2 causes the overexpression of several LINE1 transposon families, which leads to the activation of the ping-pong cycle. MILI is responsible for DNA methylation of a larger subset of transposable element families than MIWI2, suggesting that MIWI2 and MILI have independent roles in establishing DNA methylation patterns [106].

Eukaryotic DNA methyltransferases (DNMTs) are classified into DNMT1 and DNMT3. For hemi-methylated CpG dinucleotides, DNMT1 mainly functions to maintain 5mC across cell division, while DNMT3 catalyzes de novo deposition of 5mC [107]. DNMT3L can act with DNMT3a and DNMT3b methyltransferases to establish the methylation of transposons and repress their expression [108]. Under the loss of MILI or MIWI2, the methylation of retrotransposon sequences is defective at the level of de novo methylation in mice [109], while in *dnmt3L* knockout mice in which transposons fail to be methylated, the piRNAs are still produced, suggesting that piRNAs act upstream of DNMT3L to cause methylation to silence the transposable elements [110].

In human chromosomes, the piRNA clusters are dispersed in a strand-specific manner. It is unclear whether piRNAs would preferentially repress their local and proximal transposon sequences. Sigurdsson et al. have shown a significant positive correlation between the chromosomal density of hypermethylated LINE-1 and the chromosomal density of piRNA clusters, and suggested that piRNAs not only silence transposons through DNA methylation patterns, but also preferentially methylate sequences near the piRNA clusters and physically adjacent sequences on other chromosomes [111]. All of these observations suggest that piRNAs can induce DNA methylation to silence the expression of transposable elements.

The DNA methylation by the piRNA-PIWI system mainly exists in mammals to regulate chromatin structure and gene expression, whereas in *Drosophila* and *C. elegans*, piRNAs induce transcriptional repression primarily by recruiting repressive histone marks to silence the transposon expression [104].

### Histone modification

piRNAs can induce histone modification to repress the expression of transposon elements [112]. After being imported to the nucleus, the piRNA-PIWI complexes recruit histone methyltransferases and H3K9me3 onto chromatin to induce the formation of heterochromatin, thereby silencing transposon expression [4]. Huang et al. have found that repressive histone marks H3K9me2/H3K9me3 but not active histone marks are enriched at the ectopic site upon Piwi-HP1a recruitment, suggesting that the H3K9me2/H3K9me3 marks are involved in the suppression of gene expression by piRNAs [87].

In *Drosophila*, the deletion of the N-terminus from PIWI protein leads to the mis-localization of PIWI protein in the nucleus and decreases the H3K9me2/H3K9me3 marks over several transposons [113]. Moreover, Sienski et al. have shown that the loss of PIWI or the HMG (high mobility group box) protein Maelstrom, which is implicated in transposable element silencing in mice and *Drosophila*, would cause RNA polymerase II recruitment, nascent RNA output, and steady-state RNA levels of transposons [114]. The piRNA-mediated trans-silencing of transposons is likely caused by the establishment of heterochromatic H3K9me3 marks on the transposons and their genomic surroundings, for which PIWI is required [114]. Knockdown of *Piwi* results in the reduction of H3K9me3 signal in downstream target sequences while loss of *Mael* only mildly decreases transposon H3K9me3 signals [114].

The elimination of PIWI protein causes the histone marks to change on transposon sequences [115]. Thomas et al. found that the knockdown of *piwi* in the germline increased expression of transposable elements that are targeted by piRNAs in *Drosophila*. The derepression of transposons was correlated with the increased occupancy of Pol II on their promoters. Exogenously expressed piRNAs silenced the target gene, with an increase in repressive H3K9me3 marks and HP1 occupancy [116]. Furthermore, the abundance of active H3K4me2/3 marks at the target gene locus was decreased, suggesting that the piRNA-mediated transcriptional silencing correlated with the establishment of a repressive chromatin structure of H3K9me3 in the target locus [116].

In summary, piRNAs mainly degrade transposons through RNA decay, DNA methylation, and histone modification. The role of piRNAs is not limited to silencing transposons in the germline. Recent work has shown that the PIWI protein and piRNAs are expressed in somatic cells as well. Multiple lines of evidence have demonstrated that piRNAs can participate in sterility [117], tumorigenesis [118, 119], diabetes [120], cardiac hypertrophy [121], rheumatoid arthritis [122], metabolic homeostasis [6] and the aging process [123, 124]. Sun et al. have summarized some of the studies discovering abnormal expression of piRNAs in several types of cancer [125], and functions of piRNAs in the brain have also been reviewed [126–128]. Here, we mainly focus on the roles of piRNAs in neurodevelopment and neuronal diseases.

### piRNAs in neuronal tissues

While early studies have established the high abundance and functions of piRNAs in germline tissues, recent studies have demonstrated wide expression of piRNAs in somatic cells throughout the body. Although the germline tissues have the highest expression levels of piRNAs, nearly 30,000 neuronal piRNAs have been characterized in mouse brain throughout postnatal development, which suggests that piRNAs are present and dynamically regulated during brain development [129]. More detailed characterization of somatic piRNAs has shown that they are shorter in length and tissue-specific compared to germline piRNAs in mice [130]. In mice, the hippocampus in brain shows the highest abundance of piRNAs (5494 piRNAs) followed by the cortex in brain (1963 piRNAs), the kidney (580 piRNAs), and the liver (406 piRNAs), suggesting that the piRNAs expressed in different somatic tissues have different functions [130].

Apart from the role in controlling transposon levels as detailed above, piRNAs also participate in gene silencing. Increasing evidence has shown that piRNAs are expressed in the central nervous system and participate in neurodevelopment and neurotransmission [5, 11, 12]. The development of accessible and affordable small-RNA sequencing and single-cell sequencing technologies has enabled dissection and isolation of specific types of neurons and distinct brain areas with piRNA expression [131, 132]. In the following, we will review the roles of piRNAs in neuronal function (Table 3).

**Table 3:** Summary of piRNAs aberrantly expressed in neural processes and diseases

Process or disease	Category of piRNA	Mechanism	Animals/subjects	Reference
Neural development	172 piRNAs expressed	piRNAs regulate dendritic spine development	Mouse	[5]
Neural development	PIWIL1 protein	PIWIL1 promotes the multipolar morphology to the bipolar transition in cortex neurons	Mouse	[133]
Axonal regeneration	piRNA-like sncRNAs (piLRNAs)	MIWI/piLRNA complex attenuates axonal regeneration in rat sciatic nerve after crush injury	Rat	[134]
Axonal regeneration	3447 piRNAs upregulated, 4117 piRNAs downregulated	MIWI-piRNA complex participates in Schwann cell responses to nerve injury	Mouse	[135]
Axonal regeneration	piRNA pathway	piRNA pathway inhibits axonal regrowth dependent on PIWI protein PRG-1	*C. elegans*	[13]
Memory formation	Abundant expression of 28-nt piRNAs	Piwi/piRNA complex promotes serotonin-dependent methylation in the CREB2 promoter	*Aplysia*	[136]
Memory formation	PIWIL1 and PIWIL2	Knockdown of *Piwil1* and *Piwil2* enhances contextual fear memory	Mouse	[137]
Memory formation	PIWI protein less abundant in αβ mushroom body neurons	Transposon inserts in αβ neurons, including insertion into memory-relevant loci	*Drosophila*	[138]
Memory formation	piRNAs significantly changed after worm exposure to PA14	PIWI/PRG-1 is required for the induction of *daf-7* expression in ASI neurons and induces the nuclear RNAi pathway in the transgenerational inheritance of learned pathogen avoidance behavior	*C. elegans*	[139, 140]
AD	81 piRNAs upregulated, 22 piRNAs downregulated	piRNAs are nominally correlated with the genome-wide significant risk SNPs	Prefrontal cortex from AD patients	[11]
AD	146 piRNAs upregulated, 3 piRNAs downregulated	piRNA targets focus on the 5 most significant AD-associated pathways enriched with 4 genes regulated by 4 piRNAs	AD patient brain	[12]
AD	Depletion of piRNAs	Depletion of piRNAs would drive transposable element dysregulation that leads to tauopathy neurodegeneration	*Drosophila*	[15]
PD	piRNAs deregulation	Massive deregulation of piRNAs in neuronal cells	Sporadic PD- derived neuronal cells	[14]
ALS	Aub overexpression	*Caz* knockdown increases pre-piRNA levels, but reduces mature-size piRNA levels in the central nervous system	*Drosophila*	[141]
Stroke	54 piRNAs upregulated, 51 piRNAs downregulated	Transcription factors control stroke-responsive piRNAs in a redundant manner	Rat	[142]
Rett Syndrome	piRNAs increased in *Mecp2*-knockout mouse cerebellum	Upregulated transposons in Rett syndrome may lead to the increase of total piRNAs	Mouse	[143]
Neuropathic pain	piRNA-DQ541777	piR-DQ541777 recruits DNMT3a to increase methylation of *Cdk5rap1* promoter during neuropathic pain	Mouse	[144]
Anxiety	*Mili* knockout	Knockout of *Mili* induces LINE1 promoter hypomethylation in mouse brain and reduces anxiety-like behavior	Mouse	[145]

Abbreviations: *PA14 Pseudomonas aeruginosa*, *SNPs* Single nucleotide polymorphisms, *CNS* Central nervous system, *LINE1* Long interspersed nuclear elements, *DNMT3a* DNA methyltransferase 3A

### Neuronal development

piRNAs are expressed in mouse hippocampus. Lee et al., by using deep sequencing of small RNA libraries from the male mouse hippocampus, found that 0.76% of the total small RNA species matched with known piRNA sequences [5]. Among them, 172 piRNAs were detected with 5 or more tags and had a length ranging between 24 nt and 32 nt. A total of 59.3% of reads contained a “U” at the 5′ end and these piRNAs could be coimmunoprecipitated with MIWI protein, a murine homolog of PIWI, strengthening that these molecules are functional. The piRNAs combine with MIWI to form a complex. *In situ* hybridization of mouse hippocampal neurons using LNA-based anti-sense or sense probes and immunostaining with a MIWI antibody revealed that both piRNA (DQ541777) and MIWI showed a punctate pattern in the neuronal dendrite and they were co-localized throughout the neuronal dendrite. These results provide strong evidence that the piRNA-MIWI complex regulates the dendritic function in mouse hippocampus [5].

PIWI genes have also been implicated in the asymmetric division of germline stem cells in *Drosophila* [146]. Knockout of *Miwi* caused spermatogenic arrest at the start of the spermatid stage in mice [147], suggesting that PIWI is involved in spermatid polarization. In more recent work, Zhao et al. showed that PIWIL1 was expressed in the developing cerebral cortex of mice [133], and PIWIL1 and piRNAs were gradually decreased from the early embryonic stage to the neonatal stage and maintained at a low level in the brain. The newborn neurons would first go through a multipolar stage from the soma and gradually establish a bipolar morphology with one elongated neurite that leads to the migration of the neuron [148]. Knockdown of PIWIL1 resulted in a multipolar morphology of neurons and a larger number of primary neurites in neurons, suggesting that PIWIL1 promotes the transition from the multipolar stage to the bipolar stage and is indispensable for the radial migration of neurons. Furthermore, the authors found that this regulation of neuronal polarization and radial migration was partly attributed to the modulation of expression of microtubule-associated proteins by PIWIL1 in cortical neurons [133]. Taken together, these studies suggest that piRNAs regulate neuron migration and maturation during development.

### Axon regeneration

piRNAs also play a role in neuronal injury and subsequent regeneration. A decrease of MIWI protein increases axonal regrowth after injury in rats. Phay et al. have identified a subset of piRNA-like sncRNAs (piLRNAs) in rat sciatic nerve axoplasm after crush injury, which showed features of mammalian piRNAs, including a “U” at the 5' end and 2′-O-methylation at the 3' end [134]. Further, depletion of MIWI protein in mice results in increased axon growth rate, decreased axon retraction after injury, and increased axon regrowth after injury. These results suggest that the neuronal MIWI-piLRNAs attenuate axon regeneration in rats [134].

Similar results have been found in mice with sciatic nerve injury, which showed increased MIWI expression and altered piRNA expression. Sohn et al. found that 7564 piRNAs were differentially expressed after injury, among which 3447 piRNAs were upregulated and 4117 piRNAs were downregulated [135]. Transfection of an upregulated piRNA, piR-00964, downregulated the mRNA level of myelin basic protein and enhanced the migration of Schwann cells, suggesting that the MIWI-piRNA complex is involved in Schwann cell response to nerve injury [135].

In *C. elegans*, piRNA loaded into PRG-1 leads to cleavage of a target transcript or causes histone modification. Kim et al. have confirmed the presence of PRG-1 in somatic cells and that the piRNA pathway inhibits axon regeneration in *C. elegans* by affecting the post-transcriptional gene silencing [13]. They first tested whether the piRNA pathway genes were involved in adult sensory axon regeneration, and found that the loss of some factors, like PRDE-1, TOFU-3 and PRG-1, increased axon regrowth after laser axotomy. PRDE-1 and PRG-1, two essential piRNA factors, inhibited axon regeneration in a gonad-independent and cell-autonomous manner [13]. To determine whether the slicer domain of PRG-1/PIWI was required for axon regeneration, they mutated aspartate to alanine at the slicer catalytic site. The results showed that the *prg-1* mutants displayed increased axon regeneration like the *prg-1* knockout animals, suggesting that the slicer activity of PRG-1 is required for axon regeneration inhibition after injury [13].

Taken together, these results from different animal models suggest that piRNAs inhibit axon regeneration, however, the underlying mechanisms remain to be explored. It is also unclear whether piRNAs or piRNA biogenesis factors are recruited to the injured site after nerve injury or whether some specific piRNA responses participate in repair after nerve injury. These questions need to be further clarified in order to fully understand the role of piRNAs in this process.

### Memory formation

Small non-coding RNAs can cause long-lasting changes in cellular function such as memory storage. Rajasethupathy et al. have found that piRNAs are expressed extensively in neurons of *Aplysia* [136]. Knockdown of PIWI protein or specific piRNAs is sufficient to impair long-term facilitation, a type of synaptic plasticity. The PIWI/piRNA complex facilitates methylation of a conserved CpG island in the promoter of CREB2, a transcriptional repressor of memory in *Aplysia*, and therefore enhances the long-term synaptic facilitation [136].

The formation of learning and memory relies on tightly controlled gene expression patterns, and small non-coding RNAs are emerging as an indispensable regulatory mechanism. Leighton et al. knocked down *Piwil1* and *Piwil2* simultaneously in mice and demonstrated that these mice displayed greater freezing behavior after fear conditioning, suggesting that knockdown of *Piwil1* and *Piwil2* not only enhanced the long-term memory, but that this enhancement was also learning-specific [137].

Transposons can also insert into a memory-related gene to change its function. Perrat et al. have found that the mobility of retrotransposons results in genetic mosaicism in the brain [138]. In the *Drosophila* brain, transposon expression is more abundant in mushroom body αβ neurons, while the PIWI protein appears less abundant in the mushroom body αβ neurons [138]. Some transposons can insert into the memory-relevant genomic areas, and the loss of piRNAs may elevate the transposon levels in the brain, affecting the stability of memory-relevant gene expression [138].

*C. elegans* is a useful model to identify memory-related effects. The worms change their behavioral response to a conditioning stimulus paired with an unconditioned stimulus [139, 140, 149]. Recently, the Colleen T. Murphy lab has shown that piRNAs are not only related to memory formation, but also can induce transgenerational inheritance of these effects to the offspring [139, 140]. In the experimental set-up, naive worms prefer pathogenic *Pseudomonas aeruginosa* (PA14) instead of nonpathogenic *E. coli* (OP50), but after 4 h of exposure to PA14, they would switch their preference and avoid PA14 due to its virulence. Moore et al. trained the worms and found that progeny of mothers exposed to PA14 for 24 h exhibited avoidance to PA14, though they never encountered PA14 before. Remarkably, this pathogen avoidance persisted for four generations [139]. They further found that *daf-7* was more highly expressed in the ASI neurons of the progeny of PA14-trained mothers and the high level was maintained in the progeny. In addition, piRNAs were significantly changed after worm exposure to PA14, suggesting that they are involved in this behavior. The Piwi/PRG-1 complex was required for *daf-7* expression in the ASI neurons, and therefore was required for the transgenerational inheritance of the learned pathogen avoidance behavior [139]. Their most recent study showed that purified small RNAs from pathogenic PA14 were sufficient to induce pathogen avoidance in the treated worms and in four subsequent generations of progeny [140]. A single *P. aeruginosa* non-coding RNA was identified to mediate this behavior. The *prg-1* mutants were defective in the small RNA-induced avoidance response and did not have high *daf-7* expression in the ASI neurons, suggesting that piRNAs are required for the avoidance behavior in worms [140]. These studies thus provide a pathway and neuronal circuitry through which piRNAs affect this type of memory-associated behavior.

### Neurodegenerative diseases

piRNAs are overexpressed in neurodegenerative diseases. Qiu et al. have identified 9453 piRNAs in the brains of Alzheimer’s disease (AD) patients and identified 103 piRNAs that are nominally correlated with the risk of AD [11]. Among the 103 piRNAs, 81 piRNAs were upregulated and 22 were downregulated [11]. Most of the piRNAs were correlated with the genome-wide significant risk SNPs, such as *APOE* and *APOJ*, suggesting that piRNAs are a potential biomarker for AD. In another study, Roy et al. obtained similar results with 146 piRNAs upregulated in AD patients and only 3 piRNAs downregulated [12]. Results of piRNA target prediction showed that these piRNAs mainly focused on 5 most significant AD-associated pathway targets, which were enriched with 4 genes (*CYCS*, *LIN7C*, *KPNA6*, and *RAB11A*) that were regulated by 4 piRNAs (piR-38240, piR-34393, piR-40666, and piR-51810). In the two separate AD datasets, they found 10 overlapping piRNAs with differential expression, including piR-hsa-1282, piR-hsa-23538, piR-hsa-23566, piR-hsa-27400, piR-hsa-27725, piR-hsa-28116, piR-hsa-28189, piR-hsa-28390, piR-hsa-29114, and piR-hsa-7193, suggesting that some piRNAs are consistently dysregulated in AD. The overlapping piRNAs may also be biomarker candidates for AD. piRNAs are also involved in the pathogenesis of AD. Frost et al., by using *Drosophila* as a model to understand the relationship between epigenetics and tau-induced neurodegeneration [15], have found that a loss of condensation of heterochromatin and a depletion of piRNAs could cause transposable element dysregulation, which is a driver for tauopathy neurodegeneration in *Drosophila* [15].

For Parkinson’s disease (PD), massive deregulation of piRNAs has been found in sporadic PD-derived neuronal cells [14]. Schulze et al. obtained skin fibroblasts from 6 healthy controls and 9 sporadic PD patients and reprogrammed these cells to induced pluripotent stem cells. They found differential regulation of a large number of piRNAs in both cell populations and post-mortem tissue samples, suggesting that piRNAs play important roles in PD [14].

For amyotrophic lateral sclerosis (ALS), Wakisaka et al. knocked down *Cabeza* (*Caz,* a *Drosophila* homologue of *FUS*, which is one of the genes causing ALS in humans) in *Drosophila* to examine the role of Aub, a PIWI responsible for piRNA biogenesis, in neural disorders [141]. They showed that *Aub* overexpression enhanced the mobility defects caused by the neuron-specific knockdown of *Caz* in *Drosophila*. The knockdown of *Caz* increased the pre-piRNA levels but reduced the mature-size piRNAs in neurons, while overexpression of *Aub* did not increase the mature-size piRNA levels [141]. These results suggest that knockdown of *Caz* causes accumulation of pre-piRNAs, which could not be further processed by *Aub* slicers. In addition, *Aub* overexpression could also induce abnormal cytoplasmic localization of *Caz.* The above results suggested that the *Caz* knockdown-induced abnormal pre-piRNAs accumulate in the cytoplasm and contribute to the pathogenesis of ALS [141].

### Stroke

Stroke is the second most common cause of death and a main cause of disability worldwide, and has an increasing incidence due to the increasing aging population [150]. Reducing the delay to treatment is critical to maximize the benefits of reperfusion and other therapies [151]. To explore whether piRNAs are expressed in stroke, Dharap et al. profiled 39,727 piRNAs in the cerebral cortex of adult rats after transient focal ischemia and found that 54 piRNAs were upregulated and 51 piRNAs were downregulated [142]. Of the 159 transcription factors that have binding sites for piRNA gene promoters, most of them belong to 20 major families, suggesting that the transcription factors control the stroke-responsive piRNAs in a redundant manner [142].

### Rett syndrome

Rett syndrome is a severe neurodevelopmental disorder that occurs in females and leads to intellectual disability. A total of 97% of typical cases and 70% of atypical cases of Rett syndrome are caused by a mutation of the methyl CpG binding protein 2 gene (*MECP2*) [152]. Previous studies have shown that some transposons such as LINE-1 and IAPs and major satellite DNA in the nuclear fraction are overexpressed in the *Mecp2*-knockout mice [153]. To verify whether piRNAs are overexpressed in brains of *Mecp2* knockout mice, Saxena et al. measured piRNA expression levels [143]. They found that 357 piRNAs were expressed in the cerebellum, including 287 piRNAs that were more highly expressed, suggesting a global increase of piRNAs in the *Mecp2*-knockout mice. These preliminary data suggest that in Rett syndrome, the absence of *Mecp2* may upregulate transposons, which leads to the increase of total piRNAs. However, the functions of piRNAs in Rett syndrome need to be further explored [143].

### Other neuronal conditions

Zhang et al. found that a spinal piRNA-DQ541777 was significantly increased in a mouse model of neuropathic pain induced by sciatic nerve chronic constriction injury (CCI) [144]. Thermal hyperalgesia, mechanical allodynia, and spinal neuronal sensitization were reduced after knocking down the spinal piR-DQ541777 in the CCI mouse model. In addition, while CCI increased the methylation level of the promoter of *Cdk5rap1* and reduced *Cdk5rap1* expression, knocking down piR-DQ541777 reversed the *Cdk5rap1* level. These results suggest that piR-DQ541777 recruits DNMT3a to increase the methylation of *Cdk5rap1* promoter during neuropathic pain [144].

piRNAs are also involved in emotional regulation. Nandi et al. have shown the presence of small RNAs with conserved piRNA-like features in the mouse brain. Knockout of *Mili* results in hypomethylation of LINE1 promoter in mouse brains [145] and causes hyperactivity and reduced anxiety in mice. These results confirm that piRNAs exist in the mammalian brain and can contribute to brain pathology and emotional regulation [145]. All of these results suggest that piRNAs play a critical role in neuronal function and regulation. A list of studies that demonstrate aberrant expression of piRNAs in neuronal processes and diseases is provided in Table 3.

### Future perspectives

In disease studies, it remains unclear whether neurodegenerative diseases cause dysregulation of piRNAs or if piRNAs initiate a sequence of events that leads to the pathogenesis. Resolving this question remains a critical step in understanding the essential roles of piRNAs in neuronal tissues. Since piRNAs are involved in neuronal development and neurodegenerative diseases, we would wonder what the role of piRNAs in brain function is and whether they can be used as a biomarker to predict the onset of disease or used as a drug.

### piRNAs in neurodegenerative diseases

The hallmarks of neurodegenerative diseases include misfolded protein aggregation [154], lysosome and proteasomal dysfunction [155], mitochondrial dysfunction and ROS production [156], DNA damage [157], neuroinflammation [158], and neuroepigenetic transformation [159, 160]. Are piRNAs related to these processes? Based on our understanding, we summarize three functions of piRNAs that may mediate their participation in neurodegenerative processes.

First, piRNA might alter the epigenetic status in neurodegenerative diseases. Studies in AD have shown a significant change in DNA methylation of apoptotic and neuronal differentiation genes [161]. In addition, global DNA methylation is increased in late onset AD patients [162]. In a Huntington’s disease cell model, DNA hypomethylation in CpG-poor regions has been revealed [163]. These results confirm the alteration of DNA methylation in neurodegenerative diseases and further experiments are needed to confirm whether piRNAs induce the change of DNA methylation directly.

In a PD-related study, overexpression of α-synuclein in *Drosophila* and SH-SY5Y cells increases H3K9me2, which leads to gene expression changes and deregulation of synaptic activity-related genes [164]. Thus, it may be interesting to test whether the overexpressed piRNAs are linked with the aberrant epigenetic status in neurodegenerative diseases. piRNAs can recruit H3K9me2/H3K9me3 to induce heterochromatin condensation and decrease transcription. Is there any possibility that piRNAs recruit H3K9me3 to methylate and suspend the activity of Pol II to silence genes associated with synaptic function, thereby causing memory loss? Recently, Lee et al. have discovered that in sporadic AD postmortem brains, the H3K9me3-mediated heterochromatin condensation was elevated in the cortex, which further suggests  that the abnormal heterochromatin formation leads to the downregulation of synaptic function-related genes [165]. Since piRNAs are overexpressed in AD brains, are there any piRNAs that recruit H3K9me3 to form the heterochromatin and reduce synaptic function? More work is needed to confirm that the dysregulated piRNAs are related with dysregulation of histone modification and DNA methylation.

Second, piRNAs might inhibit protein degradation. Misfolded protein degradation depends upon the ubiquitin-proteasome system and the autophagy-lysosomal pathway [166]. Defects in these proteolytic systems cause aggregation of amyloid β, tau, α-synuclein, polyglutamine, and TAR DNA-binding protein 43 (TDP-43) in neurodegenerative diseases. Wakisaka et al. knocked down *Caz* to build an ALS model in *Drosophila* and found that overexpression of the PIWI protein Aub induced abnormal cytoplasmic localization of *Caz* in this model, suggesting that the abnormally expressed piRNAs might contribute to the protein aggregation [141]. Future experiments should be designed to determine how piRNAs target the proteolytic systems to damage the protein degradation process, which leads to the accumulation of misfolded proteins in neurodegenerative diseases.

Third, piRNAs might induce transposable element dysregulation. Sun et al., by using *Drosophila* and postmortem human brain samples, have found that the pathogenic tau-induced piRNA depletion promotes neuronal death through transposable element dysregulation [15]. Loss of transposons silences the neurotoxicity induced by tau in *Drosophila*. The depletion of PIWI and piRNA, and the decondensation of heterochromatin contribute to the aberrant transcription of a transposable element in tauopathy [15]. Further, increased transposable element transcripts have been found after TDP-43 knockdown in mice [167, 168], in frontotemporal dementia *Drosophila* models [169], and in AD and ALS patients [170, 171]. Based on these results, further investigations should be conducted to determine whether piRNAs cause transposon dysregulation that induces the heterochromatin decondensation, which leads to neurodegenerative diseases.

**Fig. 4:**
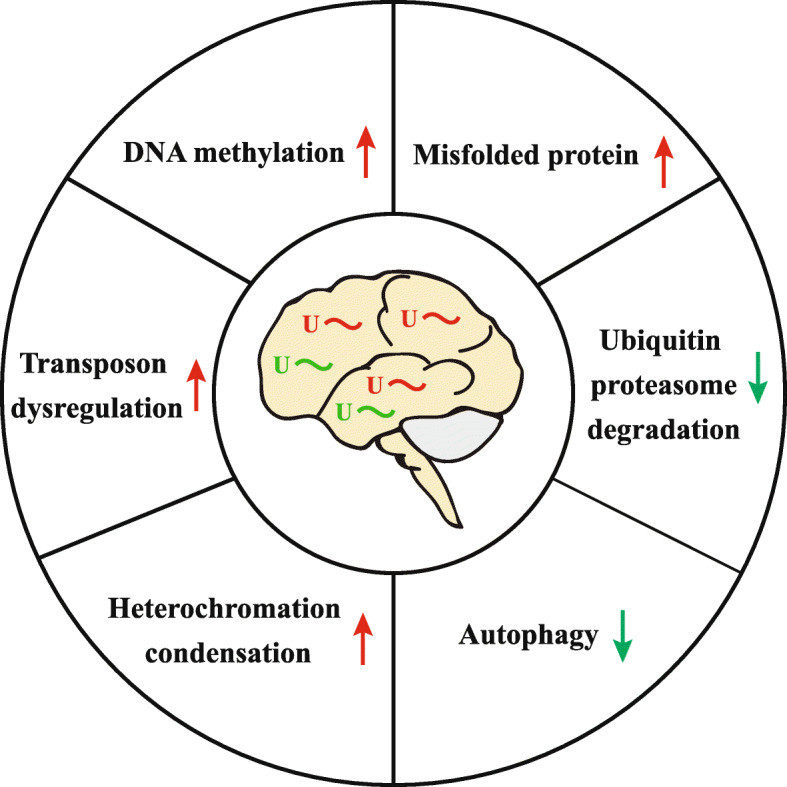
Diagram of piRNA functions in neurodegenerative diseases. piRNAs could cause transposon dysregulation, increase heterochromatin formation and DNA methylation, silence the ubiquitin-proteasome and autophagy-lysosome systems, and thus result in increased levels of misfolded protein

Taken together, the overexpressed piRNAs in neurodegenerative diseases may cause transposon dysregulation, recruit repressive factors to form heterochromatin and increase DNA methylation to silence genes associated with learning and memory. Moreover, piRNAs can cause post-transcriptional gene silencing by targeting mRNA directly to down-regulate the ubiquitin-proteasome and autophagy-lysosome systems, which increases the levels of misfolded proteins (Fig. 4).

### piRNAs act as a biomarker to predict diseases

One of the requirements for a biomarker is that samples are convenient to obtain from a patient. Fortunately, piRNAs are present in human body fluids including blood, urine and saliva [172, 173]. These findings suggest that piRNAs are  readily accessible and would be a potential prognostic biomarker for certain diseases. In addition, some of the piRNAs overlap among independent AD datasets, suggesting that they may be an indicator of AD. While it remains unclear whether the blood piRNAs are related to the neurodegenerative diseases that occur in brain, more experiments using a large number of samples should be performed to explore the changes of piRNAs in different tissues and diseases. Moreover, it is imperative to identify specific piRNAs in different stages of disease to obtain useful reference points for comparisons.

### Limitations for piRNA application

The success of Patisiran to treat the hereditary transthyretin-mediated amyloidosis based on RNAi therapy has attracted substantial attention from academic research and industry [174]. Since some diseases are caused by transposons and piRNAs can cleave mRNA directly, a question may arise as to whether piRNAs can also be used as a drug to target transposons or some mRNAs whose products cause misfolded protein aggregation.

Compared with siRNAs, which have been used in the clinic, piRNAs present new challenges. First, many piRNAs come from repetitive sequences (piRNA clusters) in the genome, therefore, they have similar sequences. Moreover, piRNAs do not require a perfect match with target RNA, where the positions 2 to 8 (seed sequence) and the positions 14 to 19 (3′ end) of piRNAs determine the piRNA-target binding and silencing activity [17]; hence, the specificity of piRNAs would be lower than siRNAs. Second, piRNAs are single-stranded precursor transcripts, while siRNAs are double-stranded, which makes them more stable than piRNAs. For possible use of piRNAs as a drug in vivo, specific chemical modifications would be needed to increase their stability. Third, piRNAs not only degrade mRNA directly, but also cause histone modification and DNA methylation, which might cause more side effects than siRNAs, due to the post-transcriptional and transcriptional gene silencing effect. piRNAs with modifications thus might also have a longer half-life and stronger effect than siRNAs. Therefore, the potential of piRNA-based therapy is worthy of more drug development efforts as well as safety and toxicity evaluations. Finally, research has shown that piRNAs can trigger transgenerational epigenetic inheritance in *C. elegans* [139, 140]. While piRNAs are mainly found in germline cells, they can easily cross through the germline and pass to soma in the next generation; therefore, the reproductive toxicity is also one of the potential risks for the use of piRNAs. Since transposons contribute to some human diseases, and piRNAs can cause powerful effects in our body, it may be worthwhile to develop piRNA-based therapies for disease treatments.

## Conclusions

piRNAs were first discovered in somatic cells almost a decade ago [175]. However, the function of piRNAs in somatic cells remains a mystery. Since piRNAs are highly expressed in the brain and dysregulated in neurological disorders, there is an impetus to evaluate the function and targets in order to understand its role in neuronal function and epigenetic regulation. Emerging and maturing concepts and technologies such as artificial intelligence, synthetic biology, and single cell sequencing will provide opportunities to decipher the neuronal functions of piRNAs. Moreover, dissection and isolation of single neurons to detect piRNA changes in different cells in distinct areas of the brain will provide another opportunity to decipher the functions. Understanding the precise function of piRNAs in neurons will facilitate the application of piRNA biology in diagnostics and/or therapies in the future.

## Data Availability

Not applicable.

## Abbreviations

AD: Alzheimer’s disease
ALS: Amyotrophic lateral sclerosis
DNMT: DNA methyltransferase
H3K9me2: Histone 3 lysine 9 di-methylation
H3K9me3: Histone 3 lysine 9 tri-methylation
LINE: Long-interspersed element
LTR: Long-terminal repeats
miRNA: MicroRNA
PD: Parkinson’s disease
piLRNA: piRNA-like sncRNA
piRNA: PIWI-interacting RNA
RDC: Rhino-Cutoff-Deadlock
RISC: RNA-induced silencing complex
TDP-43: TAR DNA-binding protein 43
USTC: Upstream sequence transcription complex
